# The Complications of Sinusitis in a Tertiary Care Hospital: Types, Patient Characteristics, and Outcomes

**DOI:** 10.1155/2015/709302

**Published:** 2015-02-02

**Authors:** Saisawat Chaiyasate, Supranee Fooanant, Niramon Navacharoen, Kannika Roongrotwattanasiri, Pongsakorn Tantilipikorn, Jayanton Patumanond

**Affiliations:** ^1^Department of Otolaryngology, Faculty of Medicine, Chiang Mai University, Chiang Mai 50000, Thailand; ^2^Department of Otorhinolaryngology, Faculty of Medicine, Siriraj Hospital, Mahidol University, Bangkok 10700, Thailand; ^3^Division of Clinical Epidemiology, Department of Community Medicine, Faculty of Medicine, Chiang Mai University, Chiang Mai 50000, Thailand; ^4^Clinical Research Center, Faculty of Medicine, Thammasat University, Pathum Thani 12120, Thailand

## Abstract

*Objective.* To study the complications of sinusitis in a referral hospital and the outcome of the treatment according to the type of complication. *Methods.* A retrospective study was performed on patients with sinusitis who were admitted to a referral hospital from 2003 to 2012. The data for the sinusitis patients who had complications were reviewed. *Results and Discussion.* Eighty-five patients were included in the study, of whom 50 were male (58.8%). Fourteen of the cases were less than 15 years old, and 27 of the patients (31.7%) had more than one type of complication. The most common complication was of the orbital type (100% in the children, 38% in the adults). After the treatment, all of the children and 45 of the adults (63.4%) recovered, eight of the adult patients died (11.3%), and 18 of the adults were cured with morbidity (25.3%). The patients with more numerous complications had poorer outcomes. When the types of complications were compared (adjusted for age, gender, and comorbidities), the intracranial complication was the only one that was statistically significant for mortality. *Conclusion.* The outcomes of the treatment depended on the number and type of complications, with the poorest results achieved in cases of intracranial complications.

## 1. Introduction

Sinusitis, which is a common ear, nose, and throat disease, develops after a viral upper respiratory tract infection in 0.5–2% of patients [1]. However, its complications are unusual. The complication rates of the patients admitted with acute sinusitis varied from 3.7 to 20% [2].

Generally, the complications of sinusitis are classified into three types: local (osseous), orbital, and intracranial complications [2, 3]. The most common complication is the orbital type (60–75%), followed by the intracranial (15–20%) and the local type (5–10%). Many studies have reported cranial nerve(s) palsy in the posterior ethmoid or sphenoiditis, which did not occur with the orbital or intracranial type [4–7]. However, optic neuropathy alone has been included in the complications of chronic sinusitis [2]. In a 1997–2002 study of Thai patients by the senior author, 8.2% of the admitted sinusitis patients had complications, but the frontal sinus was not a common cause of the intracranial complications, and cranial neuropathies did not occur with either meningitis or brain abscesses in these patients [8]. The objective of the current study was to determine the complications of sinusitis in a referral hospital and the outcome of the treatments according to the type of the complication.

## 2. Materials and Methods

A retrospective study was performed on sinusitis patients admitted to Chiang Mai University Hospital from 2003 to 2012. The data for the sinusitis patients with complications and their operative schedules were reviewed, gathered, and grouped as follows.

1. The local complications [2] included facial cellulitis, facial abscesses, osteomyelitis, and mucocele/mucopyocele that occurred either after the sinus surgery or following a previous history of sinusitis.
2. The orbital complications were classified into five groups: inflammatory oedema, orbital cellulitis, subperiosteal abscesses, orbital abscesses, and cavernous sinus thrombosis [2, 3].
3. The intracranial complications (IC) were classified into meningitis, brain abscesses (e.g., epidural and subdural), intracerebral abscesses, and dural sinus thrombosis (e.g., cavernous sinus and superior sagittal sinus) [3].
4. The authors classified cranial nerve (CN) palsy as a separate type of complication.

The data for the patients' characteristics, the organisms involved, and the outcomes of treatment were gathered. Anaerobic cultures were not available in the routine emergency setting of the hospital.

### 2.1. Statistical Analysis

The data were analysed using the STATA program version 11.0 (Stata Corporation, Texas, USA). The exact probability test was used for the proportion of the complications between the age groups, and multinomial logistic regression was used for the outcomes.

The Research Ethics Committee of the Faculty of Medicine of Chiang Mai University approved the study protocol.

## 3. Results

**Figure 1:**
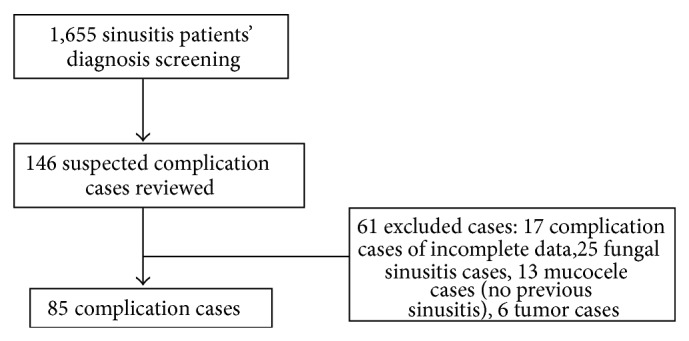
Study flow.

There were 146 suspected cases of complications in the 1,655 admitted sinusitis patients. The remainder of the patients had been admitted for sinus surgery due the failure to medically control their sinusitis. After reviewing the patients' histories, 85 patients (5.1%) were included in the study.  Figure 1 shows the 61 excluded cases, including 17 cases with incomplete data (five cases of mucocele, eight of orbital complications, one case of meningitis with an orbital complication, one of cavernous sinus thrombosis, one case with intracranial (IC) and orbital complications, and one of cerebellar abscess with a cavernous sinus thrombosis), 25 cases of fungal sinusitis, 13 cases of mucocele without a history of sinusitis (four cases had a history of a head injury and nine had no previous nasal complaints), and six tumour cases. The diagnoses of the complications were made based on the clinical findings and CT scans. Lumbar punctures and CSF examinations were performed on the patients suspected of having meningitis. All of the cases were treated empirically with intravenous antibiotics according to the organisms determined to be involved. Surgical drainage of the involved sinus, with or without the area of complication, was performed for all but one adult case with meningitis that improved with medical treatment alone.

Fifty males (58.8%) and 35 females (35%) were included in the study. Fourteen of the patients were children younger than 15 years (16.5%), and 71 were adults (83.5%). The mean age was 43.5 (±23.3), ranging from one month to 81 years. Overall, 27 of the patients had more than one type of complication (Table 1). Twenty-five of the patients (29.4%) had at least one known underlying condition that had the potential to affect their immune status and outcomes: diabetes mellitus (18.8%), chronic renal failure (8.2%), malignancy (5.9%), chronic liver disease (3.5%), and HIV infection (2.4%). The most common type of complication was orbital in nature (Table 1).

There were 15 cases of CN palsy without other types of complications. Nine of the patients had isolated unilateral or bilateral sphenoiditis, four patients had pansinusitis that also involved the sphenoid sinus, one patient had ethmoiditis, and one patient had both maxillary sinusitis and frontal sinusitis.

Of the 29 cases with local complications, facial cellulitis or an abscess was the most common complication (15 cases), followed by mucocele (12 cases) and osteomyelitis (two cases). All of the local complications except for the mucocele included the maxillary sinus with or without other sinus involvements.

In the orbital complications group (41 cases), a subperiosteal abscess was the most common complication (16 cases), followed by orbital cellulitis (10 cases), periorbital cellulitis (eight cases), cavernous sinus thrombosis (six cases), and orbital abscess (one case).

In the 24 cases of intracranial (IC) complications, five of the patients had more than one intracranial complication. The incidences of intracranial (IC) complication included 13 cases of meningitis, five brain abscesses (temporal, frontal, midbrain and pons, epidural, and along the superior sagittal sinus), and eleven cases with dural venous sinus thrombosis (eight cases of cavernous sinus thrombosis, two cases of transverse sinus and sigmoid sinus thrombosis, and one superior sagittal sinus). There were also other uncommon ICN findings, such as internal carotid artery (ICA) thrombosis, intraventricular hemorrhaging, and hydrocephalous.

The most common sinus involvement in the IC complications was the sphenoid sinus, either isolated (10 cases) or combined with the posterior ethmoid sinus (four cases). There were six cases of pansinusitis in this type of complication, three of which involved the frontal sinus alone or in combination with the ethmoid sinus and one case in which data were unavailable on the sinus involvement. Other systemic findings included sepsis, disseminated intravascular coagulation (DIC), acute respiratory failure, and liver failure.

With regard to age, all of the children had orbital complications: three with local complications and one with meningitis (Table 2).

After treatment, all of the 14 children (100%) and 45 of the adults (63.4%) fully recovered. Eight of the adult patients died (11.3%), and 18 of the adults were cured with residual morbidity (25.3%) upon hospital discharge. Of all of the cases of morbidity, those with limitations in extraocular movements recovered within two months of the follow-up period (eight cases), but the visual impairment (five cases), facial deformity/weakness (two cases) and hemiparesis (three cases) did not recover. Seven of the eight cases of mortality had intracranial complications, such as venous sinus thrombosis and meningitis with sepsis, and the other case had orbital cellulitis and sepsis. The results of the blood cultures were available for five of the eight deaths, two of which did not identify an organism and three in which the identified organisms were* Chryseobacterium indologenes*,* Staphylococcus aureus* (MRSA), and micrococcus spp.

Multinomial logistic regression was used for the analysis of the outcomes according to the number and type of complications and adjusted for age group, gender and comorbidities such as diabetes, liver disease, chronic renal disease, malignancy and HIV infection. The cases with more numerous types of complications had poorer outcomes (Table 3). Among the different types of complications, the IC complication alone had both significant morbidity (*P* = 0.042) and mortality (*P* = 0.020) (Table 4).

The pus culture reports were successfully obtained for 60 of the cases (70.1%), 24 of which showed no organisms. In the 36 cases with positive specimens, the organisms were either single or multiple, including seven cases of coagulase negative* Staphylococcus* (11.7%), five cases of* S. aureus* (8.3%), one case of methicillin-resistant* Staphylococcus aureus* (MRSA) (1.7%), seven cases of* Streptococcus* spp. (11.7%), five cases of* Pseudomonas aeruginosa* (8.3%), five cases of* Klebsiella* species (8.3%), three cases of* Enterococcus* spp. (5%), three cases of* Enterobacter* spp. (5%), three cases of* Diphtheroid bacilli* (5%), and four* Acinetobacter* spp. (6.7%), in addition to others, including* Haemophilus influenza*,* Neisseria* spp.,* Corynebacterium* spp.,* Pasteurella* spp.,* E coli, Citrobacter koseri, Proteus* spp.,* Aeromonas hydrophila,* and* Burkholderia pseudomallei*.

## 4. Discussion

Complications of sinusitis continue to occur despite the worldwide availability of antibiotics and do not always result in a complete recovery.

The results shown in Tables 1 and 2 demonstrate that the most common complication was the orbital complication, which is in accordance with the findings of the previous studies [9–12]. However, in our hospital, orbital cellulitis and subperiosteal abscesses were more commonly found than the periorbital cellulitis previously reported. This may be the result of the response to the antibiotics used in periorbital cellulitis, which improved the disease and did not require a surgical referral from other hospitals. Furthermore, in the comparison of the types of complications in the different age groups, the orbital complication was significantly more common in the children (*P* < 0.001), Table 2.

The second most common complication in this study was cranial nerve(s) palsy, followed by local complications. Other previous studies, however, have reported IC complications to be the second most common complication [2, 3]. This difference in findings may be explained by the high proportion of adults in this study, the severity of the disease, and the sinus cases that required surgical referrals, as at the beginning of our study, sphenoid sinus surgery was not performed in the other local hospitals. Table 1 shows the 15 cases that presented with CN palsy either alone or in combination with other types of complications. Potential explanations for this result may include poor hygiene, ethnicity, and the differences in the craniofacial complex and cranial base orientation, for example, the large cranial base angle in the Asian population [13–16]. As the basicranium influences the cranial shape [13], it may also affect the bone thickness and the configuration of the neurocranium as well as the facial appearance. In turn, these features may also affect the pathway for the spread of the infection and inflammation to the vasculature, bone, and cranial nerves. This suggestion is supported by the fact that sphenoid sinusitis, which has a prevalence of 1–2.7% according to the literature, is commonly observed in Asian practice, as well as in this study [5–7, 17–20]. Moreover, as in a previous study [8], the sphenoid sinus rather than the frontal sinus is the most common source of IC complications in the Thai population.

In one study in the literature, the results of treatment have been reported to vary according to the complications: 6% of the patients with IC complications died (ranging from 0 to 16%) and 23% were disabled (ranging from 0 to 46%) [21]. In our study, the overall death rate was 11.3%, while 29% of the patients with IC complications died. These higher rates may be the result of the occurrence of systemic complications such as sepsis or from the severity of the IC complication, both of which would bear monitoring and improvements with medical care.

When the types of complications were compared (adjusted for age, gender, and comorbidities), the IC complication was the only complication that was statistically significant in its poor clinical outcomes, recovery with morbidity (*P* = 0.042), and death (*P* = 0.020) (Table 4). These findings confirm those of other previous studies and should be targeted to improve the treatment outcomes in patients with complications of sinusitis.

## 5. Conclusion

The orbital complication was the most common complication in both children and adults. Additionally, in adult patients, CN palsy occurred either alone or in combination with other types of complications.

The outcomes of the treatment depended on the number and types of the complications, with the poorest results occurring in the cases with IC complications.

## Figures and Tables

**Table 1:** Type of complication from sinusitis.

Type(s) of complication	Patients (%)	Details
1	58 (68.3%)	Local: 14 9 cases of mucocele, 2 cases of facial cellulitis, 2 cases of facial abscess, and 1 case of osteomyelitis Orbital: 16 5 cases of periorbital cellulitis, 5 cases of orbital cellulitis, and 6 cases of subperiosteal abscess (SPOA) Intracranial: 13 5 cases of meningitis, 2 cases of meningitis with frontal abscess, 1 case of temporal abscess, 1 case of midbrain abscess and CN VII palsy (UMNL), and 4 cases of meningitis with other complications^*^ Cranial nerve (CN) palsy: 15 2 CN II cases 4 CN III cases 1 CN IV case 3 CN VI cases 1 CN III, CN IV case 1 CN III, CN VI case 3 CN III, CN IV, and CN VI cases

2	17 (20%)	3 mucocele cases 2 with SPOA and 1 with optic neuropathy 8 SPOA cases 4 with CN palsy (limitation of EOM all directions, visual loss) 3 with facial cellulitis/abscess 1 with osteomyelitis 4 periorbital/orbital cellulitis cases with facial cellulitis/abscess 1 orbital cellulitis and superior ophthalmic vein thrombosis with meningitis 1 transverse and sigmoid sinus thrombosis with bilateral CN VI palsy

3	8 (9.4%)	6 cavernous thrombosis cases 3 CN II, CN III, CN IV, and CN VI cases 1 CN II, VI case 1 CN III, VI case 1 CN II, VII case 1 orbital and facial abscess case with blindness 1 orbital cellulitis, scalp abscess, lid abscess, and superior sagittal sinus thrombosis case

4	2 (3%)	2 cases of cavernous sinus thrombosis with facial abscess or cellulitis

SPOA: subperiosteal abscess; UMNL: upper motor neuron lesion; CN: cranial nerve; EOM: extraocular movement.

^*^Hydrocephalus, DIC, sepsis, prevertebral abscess, and transverse and sigmoid sinus thrombosis.

**Table 2:** Types of complication classified by age groups.

Types of complication	Age <15 year (14 patients)	Age ≥15 year (71 patients)	*P* value^*^
Local (29 patients)	5 (35.7%)	24 (33.8%)	1.000
Orbital (41 patients)	14 (100%)	27 (38.0%)	<0.001
ICN (24 patients)	1 (7.1%)	23 (32.4%)	0.100
CN palsy (30 patients)	3 (21.4%)	27 (38.0%)	0.360

^*^Exact probability test.

ICN: intracranial; CN: cranial nerve.

**Table 3:** Risk (odds ratio and 95% confidence interval) of poor clinical outcomes (recovery with morbidity or death) from the total number of complication types^*^, analysed by multinomial logistic regression.

Poor clinical outcomes	OR	95% CI		*P* value
Recovery with morbidity	2.49	1.15,	5.37	**0.020**
Death	3.27	1.24,	8.63	**0.017**

Total number of complication types^*^: combined number of any type of sinusitis complication (local, orbital, intracranial complications, and cranial nerve palsy), ranging from 1 to 4.

Adjusted for age, gender, and comorbidities: diabetes, liver disease, chronic renal disease, malignancy, and HIV infection.

**Table 4:** Risk (odds ratio and 95% confidence interval) of poor clinical outcomes (recovery with morbidity or death) from sinusitis, classified by types of sinusitis complication, analysed by multinomial logistic regression.

Poor clinical outcomes and types of complication	OR	95% CI		*P* value
Recovery with morbidity				
Local	1.67	0.33,	8.40	0.534
Orbital	1.58	0.42,	5.97	0.466
IC	4.61	1.06,	20.08	**0.042**
CN palsy	3.55	0.85,	14.82	0.082
Death				
Local	1.02	0.04,	28.18	0.990
Orbital	4.82	0.15,	156.26	0.376
IC	106.55	2.06,	5512.16	**0.020**
CN palsy	0.75	0.02,	23.94	0.872

CN: cranial nerve; IC: intracranial.

Adjusted for age, gender, and comorbidities: diabetes, liver disease, chronic renal disease, malignancy, and HIV infections.
